# A New Mixed Element Method for a Class of Time-Fractional Partial Differential Equations

**DOI:** 10.1155/2014/141467

**Published:** 2014-03-09

**Authors:** Yang Liu, Hong Li, Wei Gao, Siriguleng He, Zhichao Fang

**Affiliations:** School of Mathematical Sciences, Inner Mongolia University, Hohhot 010021, China

## Abstract

A kind of new mixed element method for time-fractional partial differential equations is studied. The Caputo-fractional derivative of time direction is approximated by two-step difference method and the spatial direction is discretized by a new mixed element method, whose gradient belongs to the simple (*L* ^2^(Ω)^2^) space replacing the complex **H**(div; Ω) space. Some a priori error estimates in *L* ^2^-norm for the scalar unknown *u* and in (*L* ^2^)^2^-norm for its gradient *σ*. Moreover, we also discuss a priori error estimates in *H* ^1^-norm for the scalar unknown *u*.

## 1. Introduction

In this paper, we consider the following time-fractional partial differential equation with initial and boundary conditions

(1) $$\begin{array}{c} \frac{\partial^{\alpha }u\left(x,t\right)}{\partial t^{\alpha }}-\Delta u=f\left(x,t\right),\;\left(x,t\right)\in \Omega \times J,\\ u\left(x,t\right)=0,\;\left(x,t\right)\in \partial \Omega \times \overset{-}{J},\\ u\left(x,0\right)=u_{0}\left(x\right),\;x\in \Omega . \end{array}$$

In (1), *Ω* is a bounded convex polygonal domain in *R* ^*d*^, *d* ≤ 2 with *Lipschitz* continuous boundary ∂*Ω*, *J* = (0, *T*] is the time interval with 0 < *T* < *∞*. *u*_0_(**x**) and *f*(**x**, *t*) are given functions and ∂^*α*^ *u*(*x*, *t*)/∂*t* ^*α*^ is Caputo fractional derivative defined by

(2) $$\begin{array}{c} \frac{\partial^{\alpha }u\left(x,t\right)}{\partial t^{\alpha }}=\frac{1}{\Gamma \left(1-\alpha \right)}\overset{t}{\underset{0}{\int }}\frac{\partial u\left(x,\tau \right)}{\partial \tau }\frac{d\tau }{{\left(t-\tau \right)}^{\alpha }},\;0<\alpha <1. \end{array}$$

Fractional partial differential equations (PDEs) mainly include three types: PDEs with space fractional derivative, PDEs with time-fractional derivative, and PDEs with space-time-fractional derivative. So far, more and more people have started to pay attention to looking for the analytical and numerical solutions of fractional PDEs. In [1–14], authors proposed a lot of finite difference methods for time, space, and space-time-fractional PDEs. Lin and Xu [15] proposed and analyzed the spectral methods for solving time-fractional diffusion equation. In [16, 17], authors presented local discontinuous Galerkin methods for fractional PDEs. Li et al. [18] discussed the detailed error estimate theories of finite element methods for nonlinear space-time-fractional differential equations with subdiffusion and superdiffusion. Jiang and Ma [19] developed high-order finite element methods for one-dimensional time fractional PDE (1). In [20, 21], the finite element methods were analyzed for space fractional PDEs. In [22–24], some time-fractional PDEs were solved by the finite element methods. Zhao and Li [25] presented the fractional difference/finite element approximations for the space-time-fractional telegraph equation.

Based on the summary of the above numerical methods for solving fractional PDEs, we can see that many numerical methods, such as finite difference methods, LDG methods, finite element methods, and spectral methods, have been studied and developed. However mixed finite element methods for solving fractional PDEs have not been proposed in the current literatures.

In recent years, a lot of mixed finite element methods have been proposed by many mathematical scholars. In [26, 27], authors presented a new mixed finite element method based on the linear elliptic equations. Compared to classical mixed methods, this method has several distinct characteristics: the gradient of the new one belongs to the simple (*L* ^2^(*Ω*))^2^ space avoiding **H**(div⁡; *Ω*) space, the optimal a priori error estimates in *H* ^1^-norm for the scalar unknown *u* can be obtained, the number of total degrees of freedom for this method is less than that for classical mixed methods, and the regularity requirements on the solution *σ* = ∇*u* are reduced. In view of the method's characteristics, the new mixed method has been developed to solve some integer-order partial differential equations, such as parabolic equation [28–31], Sobolev equation [32], fourth-order parabolic equation [33], and extended Fisher-Kolmogorov equation [34].

In this paper, our aim is to study the new numerical method based on the new mixed finite element method [26, 27] for solving a class of time-fractional PDEs. We derive a new discrete method for time-fractional derivative, formulate a fully discrete mixed finite element scheme, and prove some a priori error estimates in *L* ^2^ for the scalar unknown *u* and in (*L* ^2^)^2^-norm for its gradient *σ*. What is more, we derive an a priori error estimate in *H* ^1^-norm for the scalar unknown *u*.

The layout of the paper is as follows. In Section 2, we introduce a new discrete method for the Caputo time-fractional derivative and give the ‘‘proof” of the truncation error's boundedness. In Section 3, we formulate a new mixed scheme for time-fractional PDE (1) and give the detailed proof for the a priori error estimates for two important variables based on fully discrete scheme. In Section 4, we give some remarks and extensions about the new mixed method and fractional PDEs. Throughout this paper, *C* > 0 will denote a generic constant independent of the space-time discretization parameter *h* and Δ*t*. At the same time, we denote the natural inner product in *L* ^2^(*Ω*) or (*L* ^2^(*Ω*))^2^ by (·, ·) with the corresponding norm ||·||. The other notations and definitions of Sobolev spaces as in [35, 36] are used.

## 2. Approximation of Time-Fractional Derivative

For the discretization for time-fractional derivative, let 0 = *t*_0_ < *t*_1_ < *t*_2_ < ⋯<*t*_*M*_ = *T* be a given partition of the time interval [0, *T*] with step length Δ*t* = *T*/*M* and nodes *t*_*n*_ = *n*Δ*t*, for some positive integer *M*. For a smooth function *ϕ* on [0, *T*], define *ϕ* ^*n*^ = *ϕ*(*t*_*n*_).

**Lemma 1**

The time-fractional derivative ∂^*α*^ *u*(*x*, *t*)/∂*t* ^*α*^ at *t* = *t*_*n*+1_ is approximated by the following: for 0 < *α* < 1

(3) ∂αu(x,tn+1)∂tα =Δt1−αΓ(2−α)∑k=0n[(n−k+1)1−α−(n−k)1−α]  ×3uk+1−4uk+uk−12Δt+E0n+1,

where

(4) E0n+1=1Γ(1−α)×∑k=0n∫tktk+1[(τ−tk+1)∂2u(x,tk+1)∂t2     + O((τ−tk+1)2)+O(Δt2)]   ×dτ(tn+1−τ)α.

**Proof**

As [37], using Taylor's expansion at time *t* = *t*_*k*+1_, we can arrive at

(5) $$\begin{array}{c} \frac{\partial u\left(x,t_{k+1}\right)}{\partial t}=\frac{3u^{k+1}-4u^{k}+u^{k-1}}{2\Delta t}+O\left(\Delta t^{2}\right). \end{array}$$

By (5), Taylor's expansion, and some simple calculations of definite integral, we have

(6) ∂αu(x,tn+1)∂tα  =1Γ(1−α)∑k=0n∫tktk+1∂u(x,τ)∂τdτ(tn+1−τ)α  =1Γ(1−α)∑k=0n∫tktk+1[3uk+1−4uk+uk−12Δt+∂u(x,τ)∂τ−∂u(x,tk+1)∂t+ O(Δt2)]dτ(tn+1−τ)α  =1Γ(1−α)∑k=0n3uk+1−4uk+uk−12Δt   ×∫tktk+1dτ(tn+1−τ)α+1Γ(1−α)   ×∑k=0n∫tktk+1[(τ−tk+1)∂2u(x,tk+1)∂t2+O((τ−tk+1)2)+ O(Δt2)]dτ(tn+1−τ)α  =Δt1−αΓ(2−α)∑k=0n[(n−k+1)1−α−(n−k)1−α]   ×3uk+1−4uk+uk−12Δt+E0n+1.

So, the conclusion of Lemma 1 has been arrived at by the above calculations.

**Remark 2**

In a number of studies [18, 19, 22], the time-fractional derivative with *α*  (0 < *α* < 1) order is discretized by

(7) ∂αu(x,tn+1)∂tα≈Δt1−αΓ(2−α)∑k=0n[(n−k+1)1−α− (n−k)1−α]uk+1−ukΔt.

However, the study on the discrete formulation (3) for the Caputo fractional derivative with *α*  (0 < *α* < 1) order is fairly limited.

**Lemma 3**

The truncation error *E*_0_^*n*+1^ is bounded by

(8) $$\begin{array}{c} \left|E_{0}^{n+1}\right|\leq \frac{T^{1-\alpha }}{\Gamma \left(2-\alpha \right)}\left(M_{△}\Delta t+C\Delta t^{2}\right), \end{array}$$

where *M*_△_ = sup⁡_*t*∈[0,*T*]_⁡{|∂^2^ *u*(*x*, *t*)/∂*t* ^2^|}.

**Proof**

By the simple calculations, we arrive at

(9) |E0n+1|=|1Γ(1−α) ×∑k=0n∫tktk+1[(τ−tk+1)∂2u(x,tk+1)∂t2+O((τ−tk+1)2)+ O(Δt2)]dτ(tn+1−τ)α|≤1Γ(1−α)∑k=0n∫tktk+1|τ−tk+1|×|∂2u(x,tk+1)∂t2|dτ(tn+1−τ)α+1Γ(1−α) ×∑k=0n∫tktk+1|O((τ−tk+1)2)+  O(Δt2)|dτ(tn+1−τ)α≤T1−αΓ(2−α)(M△Δt+O(Δt2)).

From (9), we can see easily that the conclusion for Lemma 3 is obtained.

## 3. New Mixed Finite Element Method

### 3.1. Mixed Formulation and Projections

In order to get the mixed scheme, we first split (1) into the following coupled system of two lower-order equations by introducing an auxiliary variable = ∇*u*:

(10) $$\begin{array}{c} \frac{\partial^{\alpha }u\left(x,t\right)}{\partial t^{\alpha }}-\nabla \cdot \sigma =f\left(x,t\right),\\ \sigma -\nabla u=0, \end{array}$$

Based on the new mixed method in [26, 27], using Green's formula, the new mixed weak formulation of (10) is to determine {*u*, *σ*}:[0, *T*] ↦ *H*_0_^1^ × (*L* ^2^(*Ω*))^2^ such that

(11a) $$\begin{array}{c} \left(\frac{\partial^{\alpha }u\left(x,t\right)}{\partial t^{\alpha }},v\right)+\left(\sigma ,\nabla v\right)=\left(f,v\right),\;\forall v\in H_{0}^{1}, \end{array}$$

(11b) $$\begin{array}{c} \left(\sigma ,w\right)-\left(\nabla u,w\right)=0,\;\forall w\in {\left(L^{2}\left(\Omega \right)\right)}^{2}. \end{array}$$

In order to formulate a new mixed finite element scheme, we first define the mixed finite element spaces. As shown in the literatures [26, 27], we choose the mixed space (*V*_*h*_, **W**_*h*_) with finite element pair *P*_1_ − *P*_0_^2^ as

(12) Vh={vh∈C0(Ω)∩H01 ∣ vh∈P1(K),∀K∈𝒦h},Wh{wh=(w1h,w2h)∈(L2(Ω))2   ∣ w2h∈P0(K),∀K∈𝒦h}.

As discussed in [26, 27], we know that (*V*_*h*_, **W**_*h*_) satisfies the so-called discrete Ladyzhenskaya-Babuska-Brezzi condition.

In view of the definition of the above mixed space, the corresponding semidiscrete mixed scheme of (11a) and (11b) is to find {*u*_*h*_, *σ*_*h*_}:[0, *T*] ↦ *V*_*h*_ × **W**_*h*_ such that

(13a) $$\begin{array}{c} \left(\frac{\partial^{\alpha }u_{h}\left(x,t\right)}{\partial t^{\alpha }},v_{h}\right)+\left(\sigma_{h},\nabla v_{h}\right)=\left(f,v_{h}\right),\;\forall v_{h}\in V_{h}, \end{array}$$

(13b) $$\begin{array}{c} \left(\sigma_{h},w_{h}\right)-\left(\nabla u_{h},w_{h}\right)=0,\;\forall w_{h}\in W_{h}. \end{array}$$

**Remark 4**

(i) If the standard mixed method is considered, the mixed weak formulation for problem (1) is to find {*u*, *σ*}:[0, *T*] ↦ *X* × **H** such that

(14a) $$\begin{array}{c} \left(\frac{\partial^{\alpha }u\left(x,t\right)}{\partial t^{\alpha }},v\right)-\left(\nabla \cdot \sigma ,v\right)=\left(f,v\right),\;\forall v\in X, \end{array}$$

(14b) $$\begin{array}{c} \left(\sigma ,w\right)+\left(u,\nabla \cdot w\right)=0,\;\forall w\in H, \end{array}$$

where *X* = {*w* ∈ *L* ^2^(*Ω*) | *w*|_∂*Ω*_ = 0}, **H** = **H**(div⁡; *Ω*) = {**v** ∈ (*L* ^2^(*Ω*))^2^ | ∇·**v** ∈ *L* ^2^(*Ω*)}.

(ii) Compared with the classical mixed weak formulation (14a) and (14b), the gradient in our scheme (11a) and (11b) belongs to the simple square integrable (*L* ^2^(*Ω*))^2^ space avoiding the use of the complex **H**(div⁡; *Ω*) space. Obviously, the regularity requirements on the solution *σ* = ∇*u* is reduced.

(iii) So far, we have not seen any related reports on the study of mixed finite element methods for solving Fractional PDEs. Here, we will give some detailed theoretical analysis on a kind of new mixed element method for solving the fractional PDE (1).

In order to analyze the convergence of the method, we first introduce two mixed elliptic projection associated with our equations.

**Lemma 5**

There exists a linear operator Π_*h*_ : (*L* ^2^(*Ω*))^2^ → **W**_*h*_ such that

(15) $$\begin{array}{c} \left(\sigma -\Pi_{h}\sigma ,\nabla v_{h}\right)=0,\;\forall v_{h}\in V_{h}, \end{array}$$

(16) $$\begin{array}{c} {||\sigma -\Pi_{h}\sigma ||}_{L^{2}(\Omega )}\leq Ch^{m}{||\sigma ||}_{m}. \end{array}$$

**Lemma 6**

There exists a linear operator *P*_*h*_ : *H*_0_^1^(*Ω*) → *V*_*h*_ such that

(17) $$\begin{array}{c} \left(\nabla \left(u-P_{h}u\right),w_{h}\right)=0,\;\forall w_{h}\in W_{h}, \end{array}$$

(18) $$\begin{array}{c} ||u-P_{h}u||+h{||u-P_{h}u||}_{1}\leq Ch^{m+1}{||u||}_{m+1}. \end{array}$$

From [26–28], we can obtain the proof for Lemmas 5 and 6.

### 3.2. A Priori Error Estimates for Fully Discrete Scheme

In the following discussion, we will analyze some a priori error estimates for fully discrete schemes based on the case 0 < *α* < 1. For the convenience of theoretical analysis, we now denote

(19) $$\begin{array}{c} B_{n-k}^{\alpha }={\left(n-k+1\right)}^{1-\alpha }-{\left(n-k\right)}^{1-\alpha },\\ D_{t}u^{k+1}=\frac{3u^{k+1}-4u^{k}+u^{k-1}}{2\Delta t}. \end{array}$$

By the discrete formula (3) for time-fractional derivative, (11a) and (11b) have the following equivalent formulation:

(20a) Δt1−αΓ(2−α)∑k=0nBn−kα(Dtuk+1,v)+(σn+1,∇v)  =(fn+1+E0n+1,v), ∀v∈H01,

(20b) (σn+1,w)−(∇un+1,w)=0, ∀w∈(L2(Ω))2.

Now, we formulate a completely discrete procedure: find (*u*_*h*_^*n*+1^, *σ*_*h*_^*n*+1^) ∈ *V*_*h*_ × **W**_*h*_, (*n* = 0,1,…, *M* − 1) such that

(21a) Δt1−αΓ(2−α)∑k=0nBn−kα(Dtuhk+1,vh)+(σhn+1,∇vh)  =(fn+1,vh), ∀vh∈Vh,

(21b) $$\begin{array}{c} \left(\sigma_{h}^{n+1},w_{h}\right)-\left(\nabla u_{h}^{n+1},w_{h}\right)=0,\;\forall w_{h}\in W_{h}. \end{array}$$

For the convenience of the analysis, we now decompose the errors as

(22) u(tn)−uhn=(u(tn)−Phun)+(Phun−uhn)=ηn+ςn;σ(tn)−σhn=(σ(tn)−Πhσn)+(Πhσn−σhn)=ρn+ξn.

Subtracting (21a) and (21b) from (20a) and (20b) and using two projections (15) and (17), we get the error equations

(23a) Δt1−αΓ(2−α)∑k=0nBn−kα(Dtςk+1,vh)+(ξn+1,∇vh)  =−Δt1−αΓ(2−α)∑k=0nBn−kα(Dtηk+1,vh)+(E0n+1,vh), ∀vh∈Vh,

(23b) $$\begin{array}{c} \left(\xi^{n+1},w_{h}\right)-\left(\nabla \varsigma^{n+1},w_{h}\right)=0,\;\forall w_{h}\in W_{h}. \end{array}$$

In the following discussion, we will derive the detailed process of proof for the fully discrete a priori error estimates.

**Theorem 7**

Supposing that *u*_*h*_^0^ = *P*_*h*_ *u*(0), *σ*_*h*_^0^ = Π_*h*_ *σ*(0); then the error estimates hold with a parameter 0 < *α* < 1:

(24a) $$\begin{array}{c} ||u\left(t_{n}\right)-u_{h}^{n}||\leq \frac{C\left(u,\alpha ,T\right)T^{\alpha }}{1-\alpha }\left(\Delta t+\Delta t^{-\alpha }h^{m+1}\right), \end{array}$$

(24b) ΔtαΓ(2−α)||σn−σhn||  ≤C(u,α,T)Tα1−α(Δt+Δt−αhm+1+hm).

**Proof**

Noting that

(25) $$\begin{array}{c} \overset{n}{\underset{k=0}{\sum }}B_{n-k}^{\alpha }\left(D_{t}\varsigma^{k+1},v_{h}\right)=\overset{n}{\underset{k=0}{\sum }}B_{k}^{\alpha }\left(D_{t}\varsigma^{n-k+1},v_{h}\right). \end{array}$$

Then (23a) may be rewritten as

(26) Δt1−αΓ(2−α)∑k=0nBkα(Dtςn−k+1,vh)+(ξn+1,∇vh)  =−Δt1−αΓ(2−α)∑k=0nBkα(Dtηn−k+1,vh)+(E0n+1,vh), ∀vh∈Vh.

We add (26) to (23b), take (*v*_*h*_, **w**_*h*_) = (*ς* ^*n*+1^, *ξ* ^*n*+1^), and multiply by 2Δ*t* ^*α*^Γ(2 − *α*) to arrive at

(27) ∑k=0nBkα(3ςn−k+1−4ςn−k+ςn−k−1,ςn+1)  +2ΔtαΓ(2−α)||ξn+1||2   =−2Δt∑k=0nBkα(Dtηn−k+1,ςn+1)+2ΔtαΓ(2−α)(E0n+1,ςn+1).

Now we consider the first term on the left-hand side of (27). Noting that *B*_0_^*α*^ = 1 and denoting *B*_*n*+1_^*α*^ = *B*_−1_^*α*^ = 0, we have

(28) ∑k=0nBkα(3ςn−k+1−4ςn−k+ςn−k−1,ςn+1)  =(3∑k=0nBkαςn−k+1−4∑k=0nBkαςn−k+∑k=0nBkαςn−k−1,ςn+1)  =(3∑k=−1n−1Bk+1αςn−k−4∑k=0nBkαςn−k+∑k=1n+1Bk−1αςn−k,ςn+1)  =3||ςn+1||2   +(∑k=0n(3Bk+1α−4Bkα+Bk−1α)ςn−k,ςn+1)   +Bnα(ς−1,ςn+1).

Substitute (28) into (27) to arrive at

(29) 3||ςn+1||2+2ΔtαΓ(2−α)||ξn+1||2=−2Δt∑k=0nBkα(Dtηn−k+1,ςn+1)+2ΔtαΓ(2−α)(E0n+1,ςn+1)−(∑k=0n(3Bk+1α−4Bkα+Bk−1α)ςn−k,ςn+1)−Bnα(ς−1,ςn+1).

For (29), we use Cauchy-Schwarz inequality to get

(30) 3||ςn+1||2+2ΔtαΓ(2−α)||ξn+1||2  ≤(∑k=0n|3Bk+1α−4Bkα+Bk−1α|||ςn−k||+Bnα||ς−1||+||Δt∑k=0n−1BkαDtηn−k−1||+ 2ΔtαΓ(2−α)||E0n+1||)||ςn+1||.

By (30), we can arrive at

(31) 3||ςn+1||2+2ΔtαΓ(2−α)||ξn+1||2  ≤C(u,α,T)Bnα(Δt1+α+Δt2+α+hm+1)||ςn+1||.

Now, we use induction to prove the conclusion (31).

*Step  1*. Setting *n* = 0 in (30) and noting that *B*_0_^*α*^ > *B*_1_^*α*^ and *B*_−1_^*α*^ = 0, we can arrive easily at

(32) 3||ς1||2+2ΔtαΓ(2−α)||ς1||2  ≤(|3B1α−4B0α+B−1α|||ς0||+B0α||ς−1||+||Δt∑k=0−1BkαDtη−1||+2ΔtαΓ(2−α)||E01||)||ς1||  =B0α(3[1−B1αB0α]||ς0||+(||ς0||+||ς−1||)+1B0α||Δt∑k=0−1BkαDtη−1||+ 2ΔtαΓ(2−α)B0α||E01||)||ς1||  ≤C(u,α,T)1B0α(Δt1+α+Δt2+α+hm+1)||ς1||.

So, when *n* = 0, (31) holds.

*Step  2*. Supposing that (31) holds, for *n* ≤ *j*,

(33) 3||ςj+1||2+2ΔtαΓ(2−α)||ξj+1||2  ≤C(u,α,T)Bjα(Δt1+α+Δt2+α+hm+1)||ςj+1||.

Now, we consider the case for *n* = *j* + 1. By (30) and the supposition (33), we have

(34) 3||ς(j+1)+1||2+2ΔtαΓ(2−α)||ξ(j+1)+1||2  ≤(∑k=0j+1|3Bk+1α−4Bkα+Bk−1α|||ςj+1−k||+Bj+1α||ς−1||+||Δt∑k=0jBkαDtηj−k||+ 2ΔtαΓ(2−α)||E0(j+1)+1||)||ς(j+1)+1||  ≤(C(u,α,T)Bj−kα(Δt1+α+Δt2+α+hm+1)×∑k=0j+1|3Bk+1α−4Bkα+Bk−1α|+Bj+1α||ς−1||+||Δt∑k=0jBkαDtηj−k||+ 2ΔtαΓ(2−α)||E0(j+1)+1||)||ς(j+1)+1||.

Noting that 1/*B*_*j*−*k*_^*α*^ < 1/*B*_*j*+1_^*α*^ in inequality (34); then we have

(35) 3||ς(j+1)+1||2+2ΔtαΓ(2−α)||ξ(j+1)+1||2  ≤C(u,α,T)Bj+1α(Δt1+α+Δt2+α+hm+1)×∑k=0j+1|3Bk+1α−4Bkα+Bk−1α|||ς(j+1)+1||.

In order to obtain the estimate for (35), we have to discuss the boundedness for ∑_*k*=0_^*j*+1^|3*B*_*k*+1_^*α*^ − 4*B*_*k*_^*α*^ + *B*_*k*−1_^*α*^|. Noting that *B*_*k*_^*α*^ > *B*_*k*+1_^*α*^, we have

(36) ∑k=0j+1|3Bk+1α−4Bkα+Bk−1α|  =∑k=0j+1|3(Bkα−Bk+1α)+(Bk−1α−Bkα)|  ≤3∑k=0j+1(Bkα−Bk+1α)+∑k=1j+1(Bk−1α−Bkα)+B0α  =5B0α−3Bj+2α−Bj+1α.

Combining (36) with (35), we arrive at

(37) 3||ς(j+1)+1||2+2ΔtαΓ(2−α)||ξ(j+1)+1||2  ≤C(u,α,T)Bj+1α(Δt1+α+Δt2+α+hm+1)||ς(j+1)+1||.

Making use of induction based on (32) and (37), we claim that (31) holds.

Note that the relationship [15] (*n*+1)^−*α*^/*B*_*n*_^*α*^ → 1/(1 − *α*) holds; then we have

(38) C(u,α,T)Bnα(Δt1+α+Δt2+α+hm+1)  =C(u,α,T)(n+1)−αBnα(n+1)αΔtα   ×(Δt+Δt2+Δt−αhm+1)  ≤C(u,α,T)Tα1−α(Δt+Δt−αhm+1).

By a substitution (38) into (31), we get

(39) ||ςn||+ΔtαΓ(2−α)||ξn|| ≤C(u,α,T)Tα1−α(Δt+Δt−αhm+1).

By a combination of (16) and (18) with triangle inequality, we get the error results of theorem.

**Theorem 8**

With the same condition, one has the following a priori error estimates:

(40) ||u(tn)−uhn||1+||σ(tn)−σhn|| ≤C(u,α,T)Tα1−α(Δt+Δt−αhm+1+hm).

**Proof**

By (21a) and (21b), we easily get

(41) Δt1−αΓ(2−α)∑k=0nBn−kα(Dtξk+1,wh) −Δt1−αΓ(2−α)∑k=0nBn−kα(∇Dtςk+1,wh)=0.

We take **w**_*h*_ = *ξ* ^*n*+1^ in (41) and *v*_*h*_ = (Δ*t* ^1−*α*^/Γ(2 − *α*))∑_*k*=0_^*n*^ *B*_*n*−*k*_^*α*^∇*D*_*t*_ *ς* ^*k*+1^ in (41) and use Cauchy-Schwarz inequality and Young's inequality to get

(42) ||Δt1−αΓ(2−α)∑k=0nBn−kα∇Dtςk+1||2  +Δt1−αΓ(2−α)∑k=0nBn−kα(Dtξk+1,ξn+1) =(−Δt1−αΓ(2−α)∑k=0nBn−kαDtηk+1+E0n+1,    Δt1−αΓ(2−α)∑k=0nBn−kα∇Dtςk+1) ≤(||−Δt1−αΓ(2−α)∑k=0nBn−kαDtηk+1||+||E0n+1||)×||Δt1−αΓ(2−α)∑k=0nBn−kα∇Dtςk+1|| ≤C(Δt2+h2m+2)+12||Δt1−αΓ(2−α)∑k=0nBn−kα∇Dtςk+1||2.

By (42), we have

(43) $$\begin{array}{c} \frac{\Delta t^{1-\alpha }}{\Gamma \left(2-\alpha \right)}\overset{n}{\underset{k=0}{\sum }}B_{n-k}^{\alpha }\left(D_{t}\xi^{k+1},\xi^{n+1}\right)\leq C\left(\Delta t^{2}+h^{2m+2}\right). \end{array}$$

By a similar discussion to Theorem 7, we get

(44) $$\begin{array}{c} ||\xi^{n}||\leq \frac{C\left(u,\alpha ,T\right)T^{\alpha }}{1-\alpha }\left(\Delta t+\Delta t^{-\alpha }h^{m+1}\right). \end{array}$$

Taking **w**_*h*_ = ∇*ς* ^*n*^ in (23b) and using (31), we arrive at

(45) $$\begin{array}{c} ||\nabla \varsigma^{n+1}||\leq ||\xi^{n+1}||\leq \frac{C\left(u,\alpha ,T\right)T^{\alpha }}{1-\alpha }\left(\Delta t+\Delta t^{-\alpha }h^{m+1}\right). \end{array}$$

Combining (44), (45), (16), and (18) with triangle inequality, we complete the proof.

**Remark 9**

It is easy to find that a priori error estimate in *H* ^1^-norm for the variable *u*, which cannot be derived based on the classical mixed scheme (14a) and (14b), is gotten.

## 4. Some Concluding Remarks and Extensions

As far as I know, the mixed finite element methods for fractional partial differential equations have not been proposed and studied. In this paper, our purpose is to present and analyze a kind of novel mixed finite element method for seeking the numerical solution of time-fractional partial differential equation with *α* (0 < *α* < 1) order derivative. We discuss two-step difference method in time direction (the approximations of the time-fractional derivative) and a class of new mixed finite element methods proposed by [26, 27] in spatial direction. We obtain some a priori error estimates in *L* ^2^ for the scalar unknown *u* and in (*L* ^2^)^2^-norm for its gradient *σ*. What is more, an a priori error estimate in *H* ^1^-norm for the scalar unknown *u* is derived, too.

In the near future, we will develop the new mixed finite element method to solve two-dimensional time-fractional Tricomi-type equations, fractional telegraph equation, and so on. At the same time, we will try to find some new approximation method for fractional derivatives and to study some other mixed finite element methods for seeking the numerical solutions of the fractional PDEs.

## References

[1] Baleanu D, Diethelm K, Scalas E, Trujillo JJ (2012). *Fractional Calculus Models and Numerical Methods*.

[2] Atangana A, Baleanu D (2013). Numerical solution of a kind of fractional parabolic equations via two difference schemes. *Abstract and Applied Analysis*.

[3] Zhuang P, Liu F, Anh V, Turner I (2009). Numerical methods for the variable-order fractional advection diffusion equation with a nonlinear source term. *SIAM Journal on Numerical Analysis*.

[4] Meerschaert MM, Tadjeran C (2004). Finite difference approximations for fractional advection-dispersion flow equations. *Journal of Computational and Applied Mathematics*.

[5] Chen C-M, Liu F, Burrage K (2008). Finite difference methods and a fourier analysis for the fractional reaction-subdiffusion equation. *Applied Mathematics and Computation*.

[6] Shen S, Liu F, Anh V (2011). Numerical approximations and solution techniques for the space-time Riesz-Caputo fractional advection-diffusion equation. *Numerical Algorithms*.

[7] Meerschaert MM, Tadjeran C (2006). Finite difference approximations for two-sided space-fractional partial differential equations. *Applied Numerical Mathematics*.

[8] Liu F, Zhuang P, Anh V, Turner I, Burrage K (2007). Stability and convergence of the difference methods for the space-time fractional advection-diffusion equation. *Applied Mathematics and Computation*.

[9] Shen S, Liu F, Anh V, Turner I, Chen J (2013). A characteristic difference method for the variable-order fractional advection-diffusion equation. *Journal of Applied Mathematics and Computing*.

[10] Wang K, Wang H (2011). A fast characteristic finite difference method for fractional advection-diffusion equations. *Advances in Water Resources*.

[11] Lin R, Liu F, Anh V, Turner I (2009). Stability and convergence of a new explicit finite-difference approximation for the variable-order nonlinear fractional diffusion equation. *Applied Mathematics and Computation*.

[12] Ashyralyev A, Dal F (2012). Finite difference and iteration methods for fractional hyperbolic partial differential equations with the Neumann condition. *Discrete Dynamics in Nature and Society*.

[13] Yuste SB (2006). Weighted average finite difference methods for fractional diffusion equations. *Journal of Computational Physics*.

[14] Sousa E (2012). A second order explicit finite difference method for the fractional advection diffusion equation. *Computers and Mathematics with Applications*.

[15] Lin Y, Xu C (2007). Finite difference/spectral approximations for the time-fractional diffusion equation. *Journal of Computational Physics*.

[16] Wei LL, He YN, Zhang XD, Wang SL (2012). Analysis of an implicit fully discrete local discontinuous Galerkin method for the time-fractional Schrödinger equation. *Finite Elements in Analysis & Design*.

[17] Wei LL, He YN, Zhang Y (2013). Numerical analysis of the fractional seventh-order KdV equation using an implicit fully discrete local discontinuous Galerkin method. *International Journal of Numerical Analysis and Modeling*.

[18] Li C, Zhao Z, Chen Y (2011). Numerical approximation of nonlinear fractional differential equations with subdiffusion and superdiffusion. *Computers and Mathematics with Applications*.

[19] Jiang Y, Ma J (2011). High-order finite element methods for time-fractional partial differential equations. *Journal of Computational and Applied Mathematics*.

[20] Zheng Y, Li C, Zhao Z (2010). A note on the finite element method for the space-fractional advection diffusion equation. *Computers and Mathematics with Applications*.

[21] Zhang H, Liu F, Anh V (2010). Galerkin finite element approximation of symmetric space-fractional partial differential equations. *Applied Mathematics and Computation*.

[22] Jiang YJ, Ma JT (2013). Moving finite element methods for time fractional partial differential equations. *Science China Mathematics*.

[23] Ford NJ, Xiao J, Yan Y (2011). A finite element method for time fractional partial differential equations. *Fractional Calculus and Applied Analysis*.

[24] Zhang XD, Huang PZ, Feng XL, Wei LL (2013). Finite element method for twodimensional time-fractional Tricomi-type equations. *Numerical Methods for Partial Differential Equations*.

[25] Zhao ZG, Li CP (2012). Fractional difference/finite element approximations for the timespace fractional telegraph equation. *Applied Mathematics and Computation*.

[26] Chen SC, Chen HR (2010). New mixed element schemes for a second-order elliptic problem. *Mathematica Numerica Sinica*.

[27] Shi F, Yu J, Li K (2011). A new stabilized mixed finite-element method for Poisson equation based on two local Gauss integrations for linear element pair. *International Journal of Computer Mathematics*.

[28] Li L, Sun P, Luo ZD (2012). A new mixed finite element scheme and error estiamtes for parabolic equations. *Acta Mathematica Scientia*.

[29] Liu Y, Li H, Gao W, He S, Fang ZC (2013). A novel characteristic expanded mixed method for reaction-convection-diffusion problems. *Journal of Applied Mathematics*.

[30] Shi DY, Tang QL (2013). A new characteristic nonconforming mixed finite element scheme for convection-dominated diffusion problem. *Journal of Applied Mathematics*.

[31] Weng ZF, Feng XL, Liu DM (2013). A fully discrete stabilized mixed finite element method for parabolic problems. *Numerical Heat Transfer A*.

[32] Shi D, Zhang Y (2011). High accuracy analysis of a new nonconforming mixed finite element scheme for Sobolev equations. *Applied Mathematics and Computation*.

[33] Liu Y, Li H, Fang ZC, He S, Wang J (2013). A coupling method of new EMFE and FE for fourth-order partial differential equation of parabolic type. *Advances in Mathematical Physics*.

[34] Wang JF, Li H, He S, Gao W, Liu Y (2013). A new linearized Crank-Nicolson mixed element scheme for the extended Fisher-Kolmogorov equation. *The Scientific World Journal*.

[35] Ciarlet PG (1978). *The Finite Element Method for Elliptic Problems*.

[36] Luo ZD (2006). *Mixed Finite Element Methods and Applications*.

[37] Liu Y, Li H, Du YW, Wang JF (2013). Explicit multistep mixed finite element method for RLW equation. *Abstract and Applied Analysis*.

